# Wind-generated Electricity in China: Decreasing Potential, Inter-annual Variability and Association with Changing Climate

**DOI:** 10.1038/s41598-017-16073-2

**Published:** 2017-11-24

**Authors:** Peter Sherman, Xinyu Chen, Michael B. McElroy

**Affiliations:** 1 Department of Earth and Planetary Sciences, Harvard University, Cambridge, Massachusetts, 02138 United States; 2 School of Engineering and Applied Sciences and Harvard China Project, Harvard University, Cambridge, Massachusetts, 02138 United States

## Abstract

China hosts the world’s largest market for wind-generated electricity. The financial return and carbon reduction benefits from wind power are sensitive to changing wind resources. Wind data derived from an assimilated meteorological database are used here to estimate what the wind generated electricity in China would have been on an hourly basis over the period 1979 to 2015 at a geographical resolution of approximately 50 km × 50 km. The analysis indicates a secular decrease in generating potential over this interval, with the largest declines observed for western Inner Mongolia (15 ± 7%) and the northern part of Gansu (17 ± 8%), two leading wind investment areas. The decrease is associated with long-term warming in the vicinity of the Siberian High (SH), correlated also with the observed secular increase in global average surface temperatures. The long-term trend is modulated by variability relating to the Pacific Decadal Oscillation (PDO) and the Arctic Oscillation (AO). A linear regression model incorporating indices for the PDO and AO, as well as the declining trend, can account for the interannual variability of wind power, suggesting that advances in long-term forecasting could be exploited to markedly improve management of future energy systems.

## Introduction

Over the past decade, China’s wind sector has expanded to the point where China now hosts the world’s largest wind energy market; the installed wind capacity in China accounted for over one third of the global total in 2015, surpassing that of the entire European Union^1^. The rise in wind power in China has been driven largely by the government’s desire to decouple energy demand from economic growth, as exemplified in the recent 13^th^ Five-Year Plan that projects an increase in installed wind capacity from 145 GW in 2015 to 210 GW by 2020^1^. Installed wind capacity is expected to reach 400 GW by 2030, equivalent to almost half of the power generating capacity from all sources currently in the US; the aggregated investment by China in wind power will amount by that time to as much as $500 billion.

**Figure 1:**
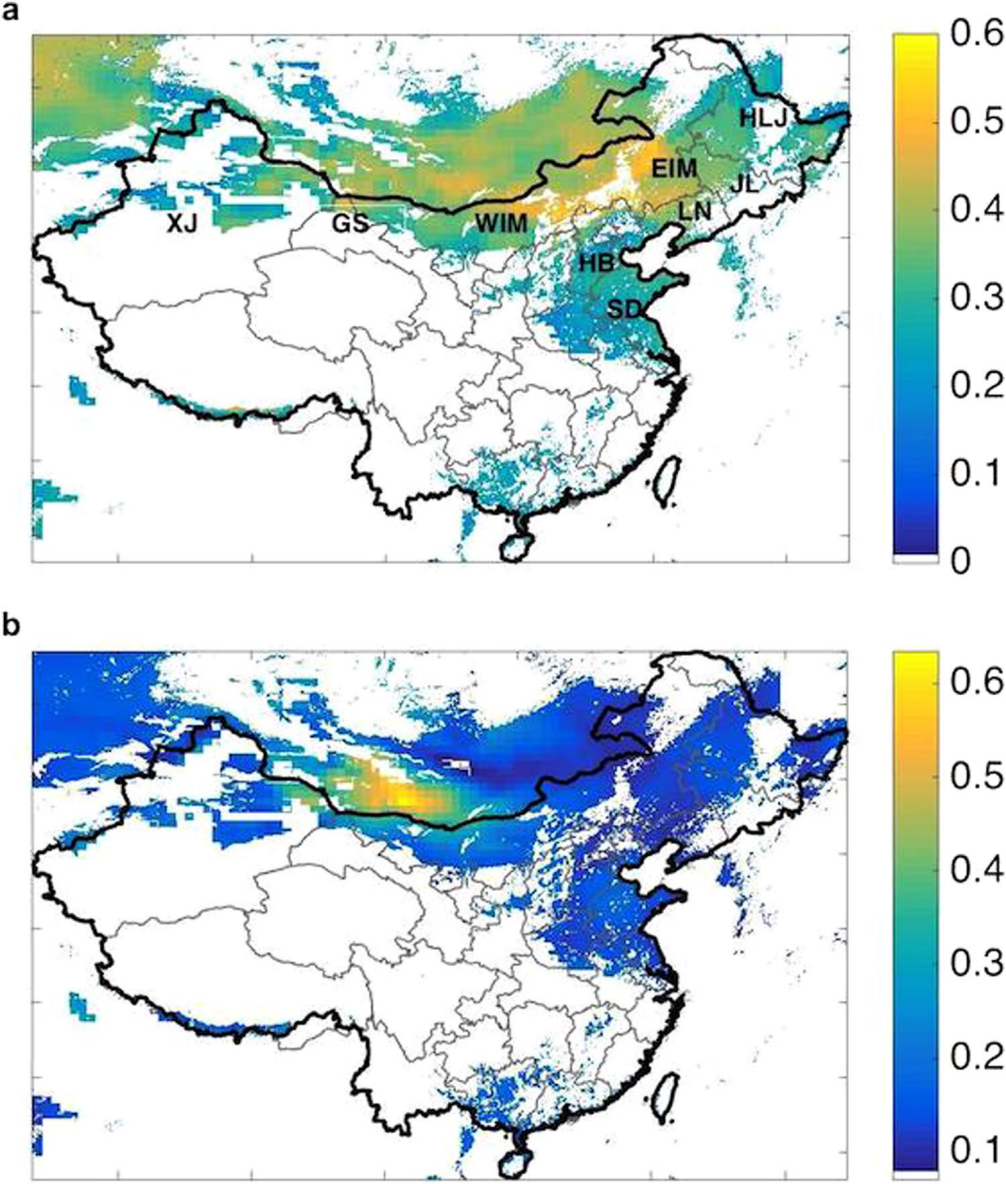
Capacity factor distribution. Distributions of the mean (panel a) and the ratio of the standard deviation (2 $\sigma$) to the mean (panel b) for capacity factors computed over the 37-year interval covered in the study. Regions indicated as blank in the figure were excluded based on at least one of the factors considered in the choice of filtering procedure. Province abbreviations are shown in panel a and explained in the Supplementary Information. (Maps are produced in Matlab R2014a).

Deployment of wind power in China is concentrated in nine wind bases (a cluster of wind farms with an aggregated capacity of more than 10 GW). Over 80% of the installed capacity is located in northern regions. On a provincial level, western Inner Mongolia (WIM), eastern Inner Mongolia (EIM), Gansu and Xinjiang lead in current installed wind capacity, accounting for 26%, 14%, 14% and 14% of the national total respectively. The above mentioned geographical regions are indicated in Fig. 1a as well as in the Supplementary Information.

The financial return and corresponding environmental benefits from investments in wind power are sensitive to long term variations and changes in wind resources. To investigate the impact of these changes, we considered a 37-year record of wind conditions covering the period January 1, 1979 to December 31, 2015. In this study, wind power is computed hourly using the power curve for GoldWind 1.5 MW wind turbines, based on hourly wind speeds available from reanalysis of meteorological data with a spatial resolution of approximately 50 km × 50 km (MERRA, as discussed in the Methods section). Areas identified as unsuitable for installations of wind turbines (such as urban areas and forests) or otherwise uneconomic are excluded in the analysis.

We find evidence for a secular decrease in the potential for wind-generated electricity for mainland China. The reduction is most significant in the north, with decreases over this studied period as great as 15 ± 7% and 17 ± 8% inferred for WIM and the northern part of Gansu, the regions targeted for the largest investments by the Chinese government. The decline in wind power potential is strongly correlated with regional winter warming in the vicinity of the SH. The strength of the SH is reported to have weakened significantly in the 1980s^2^ and the weakening of the SH is unprecedented over the past 400 years^3^. The weakening of the EAWM and SH is attributed to changes in global climate according to simulations from a number of coupled general circulation models (CGCM)^4–8^. Jiang *et al.* ^9^ ascribed the decline in wind speeds observed in winter over the past 50 years in China to a decrease in the gradient of pressure between the Asian landmass and the bordering Pacific Ocean reflecting the differential impact of climate change on temperatures over landmasses compared with the ocean.

In addition to the long-term secular decline, we find evidence for significant inter-annual variability in potential wind power, an under-appreciated source of uncertainty for power system planning and operation. This variability is attributed to fluctuations in climate associated with the Pacific Decadal Oscillation (PDO), the Arctic Oscillation (AO) and the Southern Oscillation (SO). A linear model is developed to account for the dependence of potential wind power not only on the long-term declining trend but also on the variations defined by the climate indices. Differences for individual provinces between model and data are typically less than 4%.

The PDO reflects a pattern in the Pacific Ocean characterized by two primary modes of variation in sea surface temperature (warm and cold) that shift abruptly on a multi-decadal scale^10^. The PDO index is defined by the coefficient of the first Empirical Orthogonal Function (EOF) of the mean sea surface temperature anomaly from November to March over the Pacific Ocean north of 20°N^11^. The AO reflects a similar sea surface temperature pattern for the Atlantic Ocean with an approximate decadal variability, and is observed to modulate the strength of the jet stream^12^. The AO index is defined by the coefficient of the first EOF mode of sea level pressure^13^. The Southern Oscillation (SO) identifies a further oscillation in the Pacific that persists for two to five years and is strongly correlated with El Niño events^11^. The SO index is defined in terms of the difference in atmospheric pressure between Tahiti and Darwin, Australia, with negative values corresponding to below-average wind speeds in Tahiti and above-average winds in Darwin. The correlation between the above indices and the variability of wind power in East Asia can be exploited to facilitate improved planning and operation for the overall China power sectors.

Changes in the circulation of the atmosphere over East Asia have implications not only for potential wind power but also for the regional environment more generally. A number of studies have drawn attention to the implications for air quality^14,15^, recognizing in several cases the importance of a two-way interaction between local air-quality and the circulation of the regional atmosphere. During episodes of dense haze, as occurred most recently in January of 2017 over north China, penetration of sunlight to the surface is inhibited while significant radiation is absorbed by aerosols aloft. The net effect is an increase in temperature at altitude compensated by a decrease at the surface contributing to an increase in atmospheric stability with resulting changes in circulation. A number of suggestions have been offered to account for the apparent decrease in prevailing wind speed including changes in surface roughness resulting from an increase in vegetative cover over Eurasia^16^ and a postulated expansion of the circulation of the atmospheric from the tropics^17^. Either or both of these conditions may be connected with the overall changes in climate underway globally. The present investigation highlights the fact that the decrease in potential wind generated electricity in China is significantly correlated with warming in the vicinity of the SH. While the consequences for air quality and other environmental parameters may be related to changes in average wind speed, it is important to emphasize that the implications for wind power are distinct, depending not on average values but on the cube of the wind speed. Also, the implications for potential wind power are highly localized. As indicated, the bulk of China’s wind potential is concentrated in less than 42% of its land mass.

## Results

### Wind Energy Distribution

Assimilated meteorological data on wind speeds are adopted in this paper to evaluate the wind power resources for China over the past 37 years, following the procedures described in^18–20^ and as applied for offshore environments in^21,22^. The wind database was filtered geographically so that areas identified as forested, covered with water or ice, urban or otherwise settled, characterized by slopes of more than 20% or by heights of more than 3000 meters were excluded. Areas with mean annual values for capacity factors (CFs, see Methods) of less than 15% were excluded also since wind farms placed in these regions were judged not be economically viable. In what follows, we refer to regions considered suitable for development as ‘filtered’. Expanding the time frame of previous analyses, the mean and standard deviations of CF values computed for each of the filtered grid cells covering the 37-year period and for the selected spatial domain are depicted in Fig. 1. Regions identified as blank in this figure were excluded based on the choice of filtering scheme. Peak wind energy values are observed primarily in China’s Inner Mongolia.

Compared with the analysis for Chinese wind resources in^18^, a restriction on permissible altitudes was applied, as indicated, on the basis of the geographical filter selected in the present study. This results in a significant reduction in estimates for the potential source of wind-generated power in Tibet. The threshold for acceptable capacity factors was set at 15% in the present analysis, compared with the limit of 20% assumed in^18^. The difference in this case reflects recognition of reductions in costs for investments in wind farms that have arisen in more recent years allowing systems with lower capacity factors to be profitable. The reanalysis data employed in the present study was obtained from MERRA, which provides a record of wind speeds with an hourly resolution over the past 37 years. The earlier analysis^18^ used the GEOS-5 compilation, which incorporated lower temporal resolution (6-hours) and covered a much more restricted time interval. Differences in the reanalysis data employed in the two studies combined with the differences in the selection of geographical filters and CF cut-off criteria result in a more optimistic estimate in the present analysis for the potential for wind generated power in central, eastern and southern China. Despite these differences, the two studies are in reasonable agreement as to the potential for wind-generated power for China as a whole.

Several regions are associated with high inter-annual variability. While the average variability is approximately ±17% (2 $\sigma$) in north China, inter-annual variability as great as ±41% (2 $\sigma$) is observed at locations in the western part of Inner Mongolia as illustrated in Fig. 1b. Higher inter-annual variability implies a greater risk for financial returns expected from wind project investments, in addition to higher uncertainty for power system planning and operation.

### Long-term Wind Variability

**Figure 2:**
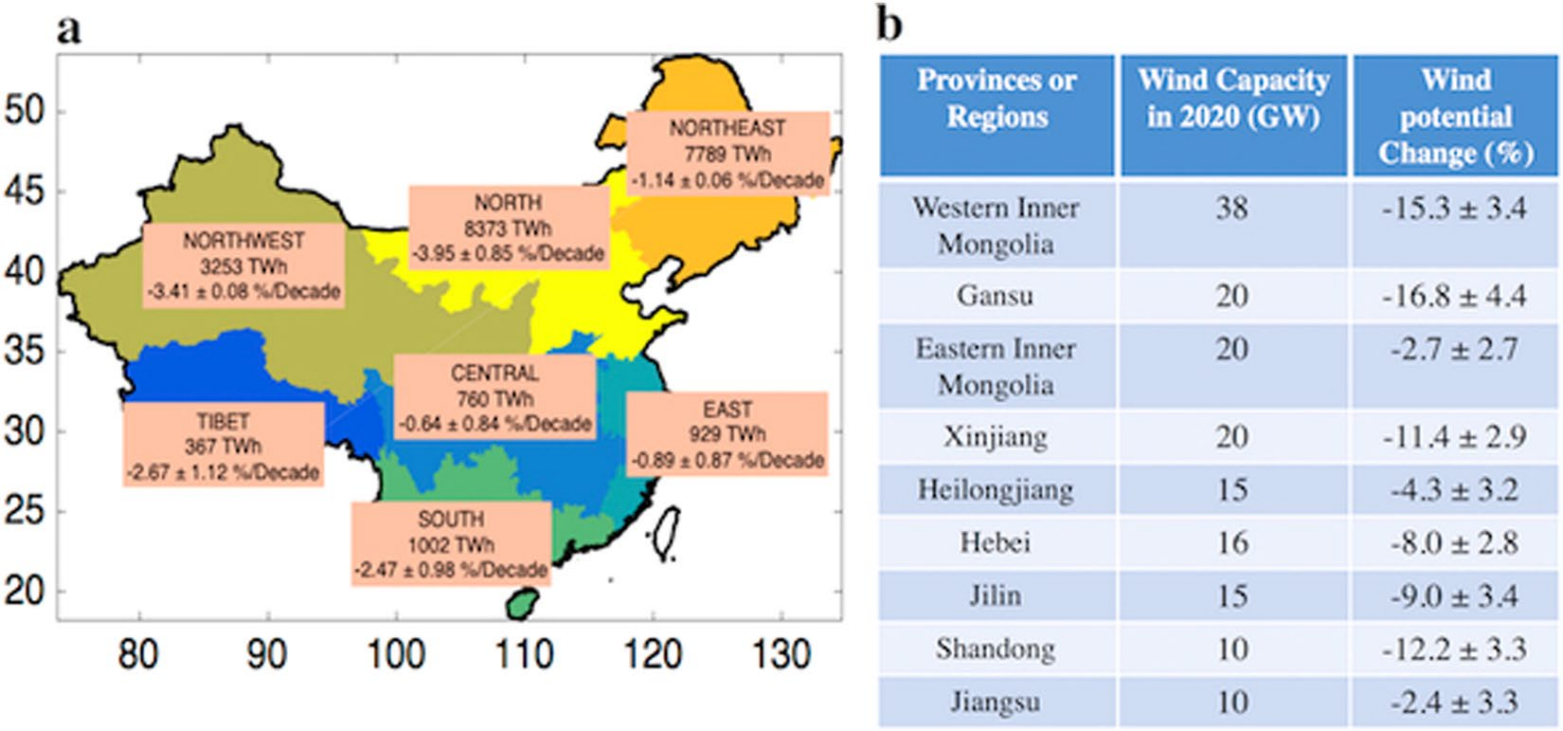
Wind potential decreasing trends. Annual means and decadal trends for the potential production of electricity from wind for different regions of China (panel a). Results are indicated in the included boxes. Table summarizing the trends in wind potential inferred over the past 37 years together with capacities projected for the major sites in 2020 (panel b). (The map is produced in Matlab R2014a).

The 37-year average yield for electricity that could be produced from wind was calculated for each region with results displayed in Fig. 2. The vast majority (86%) of the potential electricity source, as expected, is located in the northern regions. However, these are also the environments projected to have undergone the greatest decline in potential wind generated electricity over the time interval covered in the study. The decline is particularly notable in the northern part of Gansu, in western Inner Mongolia and in Shandong, regions of high power potential (and targets for major future investments) that have exhibited drops in potential over the past 37 years of 17%, 15% and 12% respectively, as indicated in Fig. 2b. For the onshore wind bases in Jiangsu, Heilongjiang and EIM, the trends indicated here are judged not to be statistically significant.

Wind energy data were partitioned into four bins defined by varying CFs with results plotted as time series over the selected time interval covering the entirety of the filtered regions (Fig. 1). While there is a noticeable decline in the trend for all wind conditions (consistent with observations in^23^), the decline is greatest at highest wind speeds; the CF value corresponding to the largest CF bin (CF > 0.35) decreases by 3.57 ± 0.48%/decade, nearly six times greater than the 0.61 ± 0.51%/decade decrease obtained for the smallest bin (0.15 < CF < 0.225). The trend indicates that the optimal locations for siting of wind farms may have become notably less productive over the past four decades.

**Figure 3:**
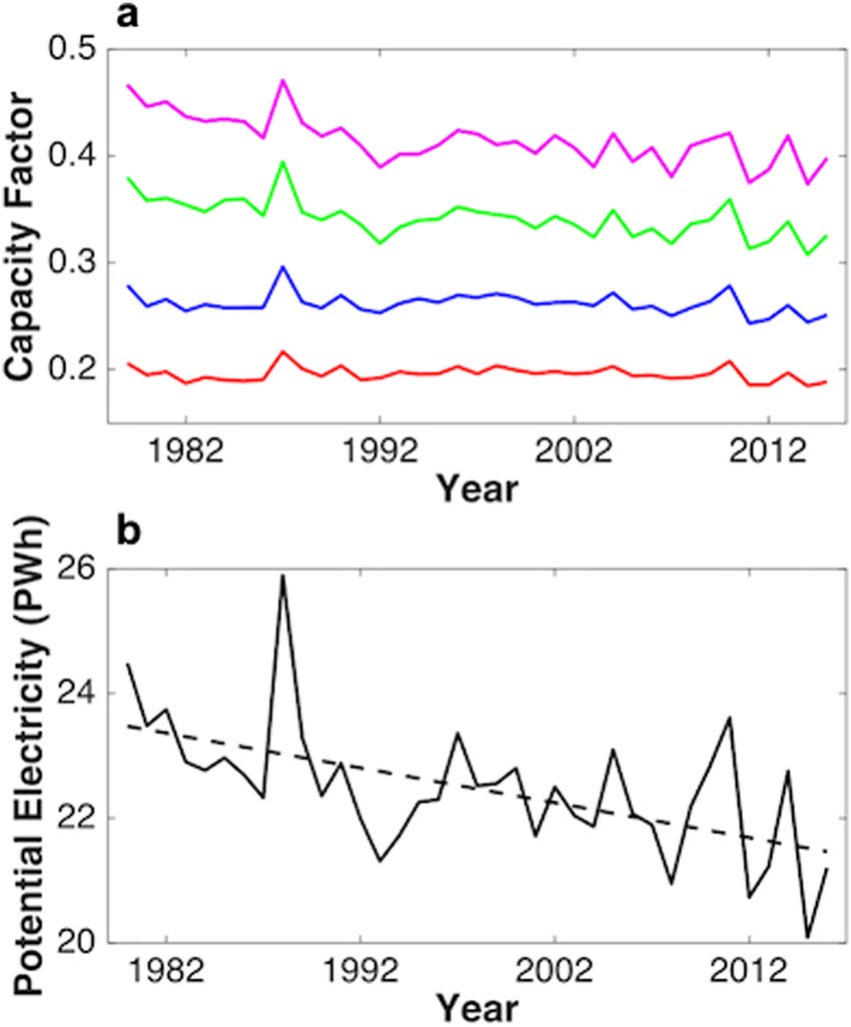
Capacity factor time series. Time series of annual capacity factors computed for the selected region covering the 37-year extent of the study (panel a). Capacity factors were divided into four groups: 0.15 to 0.225 (red), 0.225 to 0.3 (blue), 0.3 to 0.375 (green) and greater than 0.375 (magenta). A time series for total potential electricity generation, including a linear fit to these data is displayed in panel b.

A linear trend line was fitted to the annual source of electricity estimated for Mainland China as a whole, as displayed in Fig. 3. The data summarized here provide convincing evidence for a statistically significant decline (p = 2.5 × 10^−4^) in electricity that could have been generated from wind in China over the past 37 years (a decrease of approximately 13% between 1979 and 2015). The declining trend of wind speed is similar with different reanalysis products (as indicated in the Supplementary Information) and consistent with the reported observations^8,16^.

**Figure 4:**
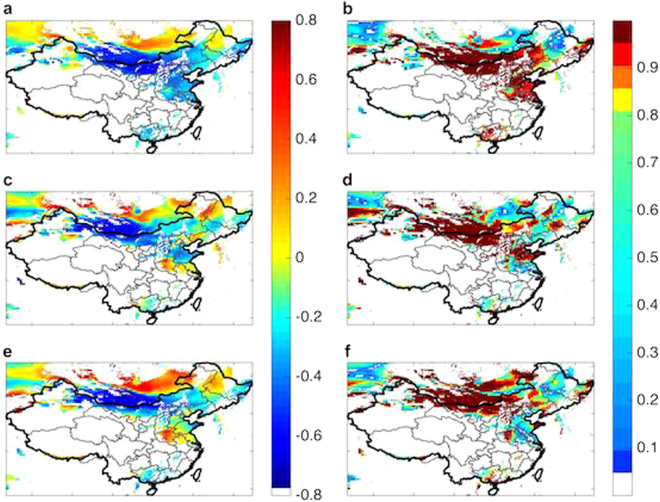
Capacity factor-temperature correlations. Correlations between CF and surface temperature over the selected region as a function of the choice of filtered locations for the 37-year interval covered in the study: (**a**) correlation with the SH annual average surface temperature; (**c**) correlation with the global mean surface temperature anomaly; (**e**) correlation with local annual average surface temperature. The statistical significances are shown in (**b**,**d**) and (**f**) for their respective correlation maps. (Maps are produced in Matlab R2014a).

The annual production of electricity from wind for major wind bases in China is dominated by contributions during winter and spring. The average capacity factor for March and April is 44% higher than that for the summer months of July and August, as indicated in the Supplementary Information. The wind power for winter and spring is determined by properties of the East Asia Winter Monsoon (EAWM), modulated mainly by the strength of the SH. A strong negative correlation is observed between local wind potential and average temperatures in the critical Siberian region (40–65°N, 80–120° E), with correlation coefficients as great as −0.72 ± 0.12 in WIM as indicated in Fig. 4a. The regions identified as statistically significant, as displayed in Fig. 4b, include most of northern and northwestern China covering seven of the nine existing wind bases accounting for 45% of total wind power potential available for China as a whole. The SH has exhibited a notable shift eastward over the past two decades^14^ in addition to a reduction in its strength since the 1980s – a change that appears to be unprecedented over at least the past 400 years^3^. The weakening of the SH is associated also with an increase in local temperatures^2^, accounting for the strong negative correlation between average temperatures in the vicinity of the SH and the variation of potential wind power inferred here.

The potential for wind power is correlated further with both global average and local temperatures as indicated in Fig. 4c and e. The correlation between global average surface temperatures and wind power potential inferred for the WIM reaches values of −0.62 ± 0.20 in WIM. As indicated in Fig. 4d, this correlation is statistically significant for Xinjiang, Inner Mongolia, Gansu, Shandong, Hebei and parts of the northeastern China, which jointly cover the majority of installed wind bases in China. The strong correlation between global climate and wind power in China, as will be discussed later, is attributed mainly to changes in the properties of the EAWM. The change inferred for the past 37 years has been associated not only with a decrease in wind potential but also with a rise in temperature. Warmer winter temperatures in northern China will result most likely in a decrease in demand for residential heat, a resource supplied significantly in these regions by coal-fired combined heat and power plants. The reduction in emissions of CO_2_ from these plants could compensate at least in part for the reduction in the potential supply of power from wind.

### Correlations with Sources of Natural Climatic Variability

**Figure 5:**
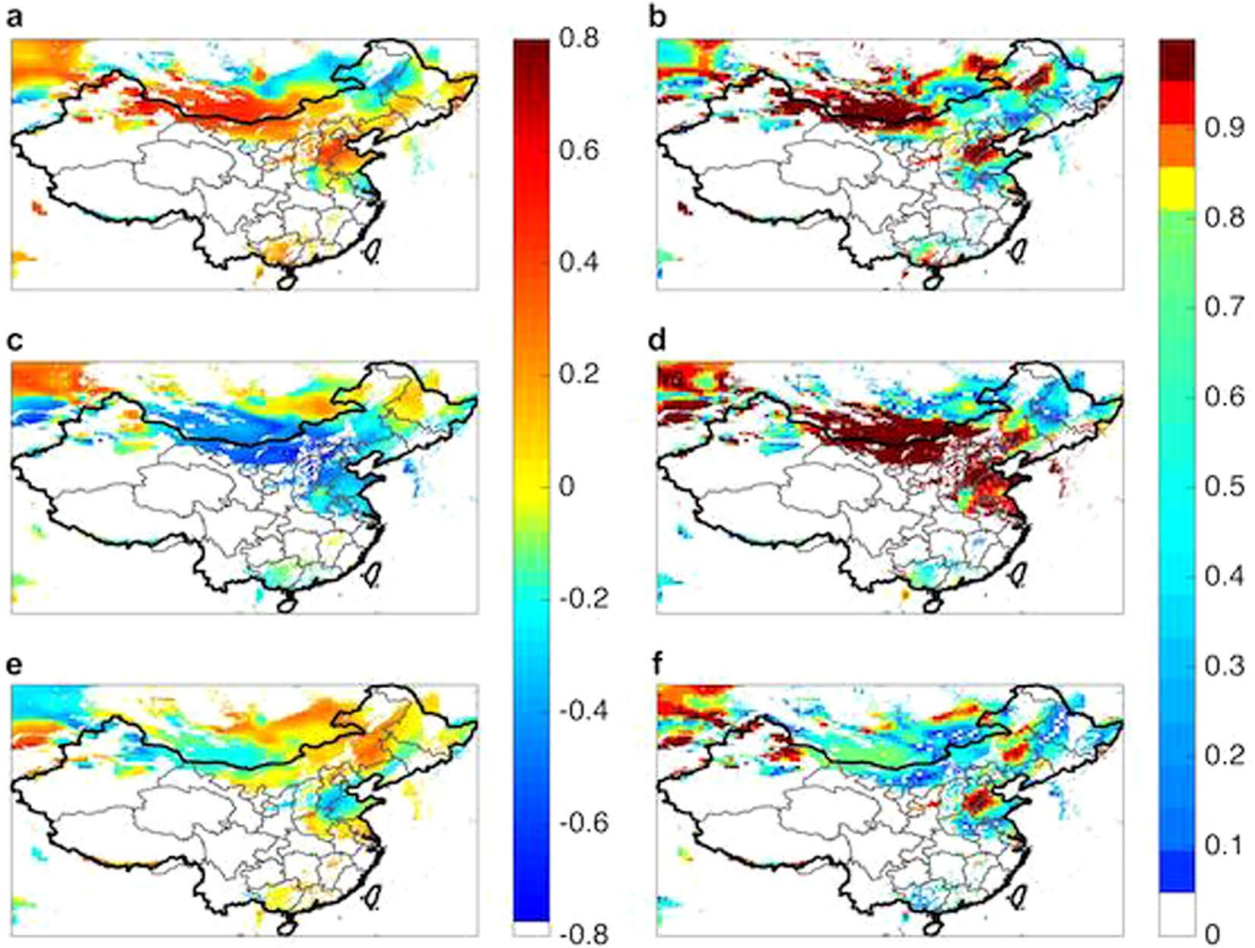
Capacity factor-climate index correlations. Distributions of correlations between CF and the PDO (**a**), AO (**c**) and SO (**e**) over the analyzed region with filtered locations as discussed for the entire 37-year interval. The statistical significances are shown in (**b**), (**d**) and (**f**) for their respective correlation maps. (Maps are produced in Matlab R2014a).

Correlations between CF and parameters identifying selected variable oscillatory properties of the global climate are presented in panels a, c and e on Fig. 5. The statistical significance of these correlations is discussed in panels b, d and f of the figure. A strong positive correlation with the PDO is observed over the western portion of Inner Mongolia, in Xinjiang, in the northern part of Gansu, and in Hebei. The correlation with the AO is distinctly negative over these regions. The correlation with the SO is negative over Shandong and over coastal Hebei but is otherwise the weakest of the three associations considered. The behavior observed here is consistent with current understanding of the relevant physics.

The strengths of the Aleutian Low and Mongolian High are enhanced during the warm (positive) phase of the PDO with their locations shifted towards central China. This results in formation of a region of anomalously high pressure over central eastern China conducive to the development of strong winds^24,25^ consistent with the pattern of the correlations depicted in Fig. 5a.

The AO is known to play a significant role in modulating properties of the EAWM in winter. A positive (negative) AO index is associated with strong (weak) westerly winds over northern Eurasia, with weakening (strengthening) of the East Asian Trough, resulting in higher (lower) temperatures over East Asia and a weak (strong) EAWM^26^. From this reasoning, we would expect a strong negative correlation between CF and AO over northern China, precisely the condition depicted in Fig. 5c.

Chen *et al.* ^27^ indicate that a weak (i.e. negative) ENSO phase is associated with an anomalous anticyclone over the western North Pacific in winter leading to enhancement of southern winds over the Chinese coast during the following summer. Theory indicates therefore that there should be a strong negative correlation between CF and the phase of the ENSO signal along the coast. This expectation is consistent with the results displayed in Fig. 5e.

### Multi-variable regression model for the inter-annual variability of wind power

Given the significance of the correlations of CF with the individual climate indices displayed in Fig. 5, we were encouraged to develop a representation for the relationship between CF values and a composite version of these indices. To this end, we chose to focus on the variability of CF values with respect to the underlying long-term decline inferred both for individual regions and for the environment studied as a whole. Annual average values of CF were fitted in terms of linear combinations of a variable representing time and indices describing the variability of the climate system associated with the PDO, AO, and SO. The linear regression models incorporating single climate indices and possible combinations of these indices are compared using the Akaike Information Criterion (AIC), as described in the Supplementary Information. The AIC criterion is designed to assess the statistical significance of different statistical models, to determine the optimal combination of input variables and to exclude the possibility of over-fitting or employment of redundant or self-correlated inputs. The linear regression model incorporating indices defining the AO and PDO performs best for Xinjiang, while the regression model incorporating only the AO index provides the best representation for Hebei and WIM. The AIC criterion fails to support the SO as a significant contributor to the variability of wind power over the studied regions, and the SO is therefore excluded from the regression modelling. Details of the procedure employed here are outlined in the Methods section below. Optimal regression models and corresponding regression coefficients are summarized in the Supplementary Information.

**Figure 6:**
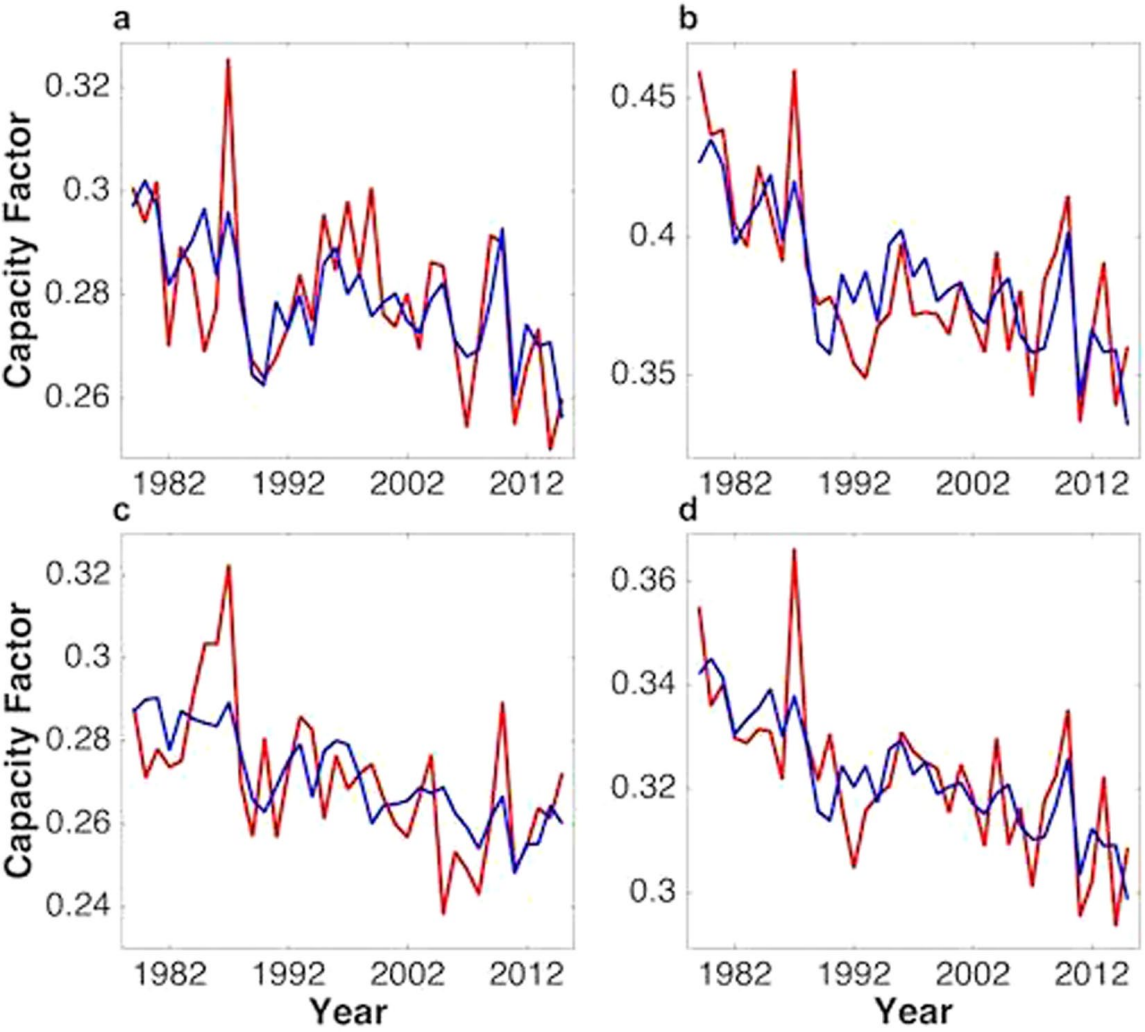
Multivariable regression modelling. Time series, represented in red, of CF values for Hebei (panel a), western Inner Mongolia (panel b), Xinjiang (panel c) and for the entirety of the analyzed region (panel d). Results obtained using the regression model are indicated in blue.

Results obtained using this procedure to represent the trends in CF obtained for Hebei, western Inner Mongolia, Xinjiang, and for the region as a whole are presented in Fig. 6. The procedure provides an excellent representation of the trends observed for CF with maximum differences between the linear representations and actual data as low as 3% (Hebei, panel a), 3% (western Inner Mongolia, panel b), 4% (Xinjiang, panel c), and 2% for the region as a whole (panel d). The r^2^ values are as high as 62% and 57% for western Inner Mongolia and for the entire region, as summarized in SI. This suggests that the indices could be employed usefully to calculate CF values over the regions reflecting this strong correlation. We should note that the regression model incorporating the AO and PDO indices provides a good fit to the de-trended inter-annual variation of wind power, as well. The r^2^ value is 0.42 for the entire region. The corresponding result is indicated in the Supplementary Information.

## Discussion and Conclusions

The main purpose of this study was to analyze the temporal variability of electricity that could have been produced from wind in China over the past 37 years. We highlighted the fact that there has been a noticeable decline in the potential for available wind power, especially in areas with otherwise superior conditions. Wind power potential declined most significantly in regions identified with the largest investment in wind systems projected for 2020, including western Inner Mongolia and the northern part of Gansu. In contrast, the wind power potential for Jiangsu (coastal wind base) is relatively stable (no statistically significant decline). The regional allocation for future wind investments might need to shift in response to projections for future changes in wind resources.

As indicated earlier, the variability of power production from wind in China is attributed primarily to variations in the characteristics of the EAWM, modulated primarily by the strength of the SH. The strength of the SH has weakened significantly since the 1980s based on monthly winter pressure maps and observations^2^ and tree ring data^3^. It has been argued that the unprecedented weakening of the SH and EAWM is associated with large-scale, on-going, changes in global climate^4–9^. Hu *et al.* ^4^ used a coupled land-ocean GCM model to simulate different climate scenarios and concluded that increases in greenhouse gas concentration resulted in a decrease in the intensity of the East Asian Winter Monsoon. The simulation results are consistent with conclusions from other modeling simulations incorporating multiple CGCM models^5–8^. The studies indicate that changes in global climate would have had an important negative influence on the economics of wind investments in China over the recent past and could have a consequential negative influence in the future.

With the increasing contribution of electricity from wind and solar, the operation and planning of the power system are subject to increased uncertainties associated with variability in climate. The inter-annual variation of wind power will influence not only the revenue stream for the wind producer, but will have an impact also on fuel consumption, on the revenue for thermal units and on the utilization rates for some critical transmission corridors. In the power system planning process, the capacity anticipated for thermal power plants is projected based on forecasts for peak power demand, accounting also for contributions anticipated from wind power, referred to as the capacity credit^28^. The capacity credit is assessed currently based on information available from records for wind power production available for an interval of between one and three years of past experience. The analysis depends thus largely on the choice of years for which these data are acquired, leading to potential bias in investments for projected future thermal capacity. With the strong links between climate variability and wind power identified here, the variation and uncertainty of wind power could be better defined.

Under the current vertically integrated electricity system in China, a significant portion of the operation of the power generation system is planned on an annual basis. The annual quota for individual generators is determined at the beginning of the year. A better forecast of the potential for annually wind generated electricity could enhance significantly this annual planning process. For an electricity market, the long term wind forecast information could improve decisions with respect to bilateral contracts between energy producers and consumers, while at the same time facilitating bidding of financial transmission rights, pricing of renewable energy credits and other financial options in the power market, all of which will be increasingly important at projected future elevated levels of contributions from renewable sources. Wind power forecasts, however, are currently effective mainly on a time scale of less than 72 hours^29^, limited largely by the stochastic nature of weather forecasts. There are suggestions that seasonal and annual forecasts for climate oscillations may become increasingly reliable in the future^30–32^. The PDO, for example, may be predictable potentially for as much as two to four seasons in advance^33^. The regression model developed here connects long term variations of wind power and climate oscillations, leading to possibilities for improving the annual and seasonal wind forecasts. A variety of advanced mathematical models could be adopted for the development of actual wind forecast software, such as the ARIMA model^34^, an artificial neural network supporting vector machines and their possible combinations.

On a longer time horizon, the predicted tendencies and uncertainties of the PDO, AO and SO indices could be useful in characterizing the uncertainty of wind power potential. Lapp *et al.* ^35^ project that the PDO will tend “towards more occurrences of the negative phase PDO” over the next century. It is difficult to distinguish between changes in the AO and anthropogenically prompted changes in climate, but Visbeck *et al.* ^36^ expect the positive phase to become more prevalent in the future. Uncertainty associated with future SO index predictions is discussed by Cane^37^. Extension of the approach advanced by Pryor and Barthelmie^38^ to model anticipated future changes in response to ongoing anthropogenic climate change as well as natural climate oscillations could also be valuable, and could make an important contribution to long- term power system planning.

## Methods

The wind data employed in this study were taken from MERRA, a NASA reanalysis product using a new version of the Goddard Earth Observing System Data Assimilation System Version 5 (GEOS-5)^39^. The wind speed data are publicly available from the NASA database. This database defines hourly wind speeds with a spatial resolution of 1/2 degree longitude by 2/3 degree latitude from 1979 to present. Hourly wind speeds at 80 m were extrapolated from 10 m and 50 m using the vertical profile of the power law described by Archer and Jacobson^40^. Instead of assuming a value of 1/7 for the associated friction coefficient, we evaluated the friction coefficient in this analysis using wind speeds represented at 10 and 50 m for each grid cell, as in Lu *et al*.^41^. Wind power was computed hourly using the power curve for GoldWind 1.5 MW wind turbines, a typical system deployed for onshore applications in China^42^. The ratio of real hourly power output to the nameplate capacity of turbines was used to compute the hourly capacity factors (CFs).

The geographical information is incorporated then to determine feasible regions for deployment of wind power systems, as described in the main text. The geographical information employed to determine the land coverage characteristics was derived from a global land use database^43^; elevation data used to determine altitudes and slopes were acquired from^44^.

To determine the maximum possible wind power generated over Mainland China, we needed to specify an appropriate areal spacing for turbines. Davidson *et al.* ^45^ determined that in order to minimize turbine-turbine interference, turbines should be separated by approximately 9 × 9 rotor diameters (0.58 km^2^). The area of an individual coordinate cell was divided thus by this value to compute the number of turbines that could fit maximally into a given cell. The potential electricity that could be generated in each cell was calculated taking these considerations into account.

In studying the correlation between estimated wind and global mean temperatures, we used mean monthly land surface temperature anomaly data from the Goddard Institute for Space Studies (GISS) Surface Temperature Analysis, which utilizes meteorological observations covering much of the Earth^46,47^. This dataset extended over the full 37-year duration of the present study. In studying the correlation between wind potential and local temperatures we used monthly land and ocean surface temperature anomaly data from the Berkeley Earth Surface Temperature Study, which utilizes observations from over 39,000 stations covering much of the Earth^48^. This dataset has a spatial resolution of 1 degree longitude by 1 degree latitude and extended over the full 37-year extent of this study.

Monthly climate indices for the PDO, AO and SO were taken from databases maintained by NOAA^49–51^ and are available also for the entire duration of the time interval covered here.

Temperature anomalies were calculated for each year, defining *I*_*TEMP*_, a 37 × 1 vector. Similarly, the mean CF for every year was computed to form *CF*_*YEAR*_, also with dimension 37 × 1. We calculated then the correlation between *I*_*TEMP*_ and *CF*_*YEAR*_ for each coordinate cell in the region of interest. The correlation coefficient for every coordinate cell was then mapped. The same correlation process was performed for each of the indices *I*_*PDO*_, *I*_*AO*_ and *I*_*SO*_ and *CF*_*YEAR*_, with results as indicated in Fig. 5.

A representation for CF, *CF*_*rep*_, was developed in terms of a linear combination of *I*_*PDO*_, *I*_*AO*_, *I*_*SO*_ and time *t* expressed as

1 $$C{F}_{rep}=\,{a}_{1}\,+\,{a}_{2}{I}_{PDO}\,+\,{a}_{3}{I}_{AO}\,+\,{a}_{4}{I}_{SO}+{a}_{5}t,$$

where *a*_1_, …*a*_5_ represent coefficients determined through an optimal fit to the derived fields of CF. This approach was applied to the entire region of interest, as well as to specific provinces. Values for *a*_1_, …*a*_5_ were obtained by applying the multivariable regression procedure to time series of CF calculated for Hebei, Western Inner Mongolia, and Xinjiang as well as for the region as a whole. The Akaike information criterion (AIC) was applied to determine the most effective combination of input variables, with model results displayed in Fig. 6. Detailed procedures and values for the relevant coefficients are summarized in the SI. All statistical analyses were conducted based on the statistical toolbox in MATLAB.

## Electronic supplementary material

Supplementary Information

## References

[1] International Energy Agency, China 13^th^ Wind EnergyDevelopment Five Year Plan (2016–2020), https://www.iea.org/policiesandmeasures/pams/china/name-161251-en.php?s=dHlwZT1yZSZzdGF0dXM9T2s,&return = PG5hdiBpZD0iYnJlYWRjcnVtYiI-PGEgaHJlZj0iLyI-SG9tZTwvYT4gJnJhcXVvOyA8YSBocmVmPSIvcG9saWNpZXNhbmRtZWFzdXJlcy8iPlBvbGljaWVzIGFuZCBNZWFzdXJlczwvYT4gJnJhcXVvOyA8YSBocmVmPSIvcG9saWNpZXNhbmRtZWFzdXJlcy9yZW5ld2FibGVlbmVyZ3kvIj5SZW5ld2FibGUgRW5lcmd5PC9hPjwvbmF2Pg, (2017), (Date of access: 10/06/2017).

[2] Panagiotopoulos F, Shahgedanova M, Hannachi A (2005). & Stephenson, D. Observed Trends and Teleconnections of the Siberian High: A Recently Declining Center of Action. Journal of Climate.

[3] D’Arrigo R, Jacoby G, Wilson R, Panagiotopoulos F (2005). A reconstructed Siberian High index since A.D. 1599 from Eurasian and North American tree rings. Geophys. Res. Lett..

[4] Hu Z, Bengtsson L, Arpe K (2000). Impact of global warming on the Asian winter monsoon in a coupled GCM. Journal of Geophysical Research: Atmospheres.

[5] Hori ME, Ueda H (2006). Impact of global warming on the East Asian winter monsoon as revealed by nine coupled atmosphere-ocean GCMs. Geophysical Research Letters.

[6] Kimoto M (2005). Simulated change of the East Asian circulation under global warming scenario. Geophysical Research Letters.

[7] Hu ZZ, Yang S, Wu R (2003). Long-term climate variations in China and global warming signals. Journal of Geophysical Research: Atmospheres.

[8] Jiang Y, Luo Y, Zhao Z (2010). Projection of wind speed changes in China in the 21^st^ century by climate models. Chinese Journal of Atmospheric Sciences (in Chinese).

[9] Jiang Y, Luo Y, Zhao Z, Tao S (2009). Changes in wind speed over China during 1956–2004. Theoretical and Applied Climatology.

[10] Aguado, E. & Burt, J. Understanding Weather and Climate – 2^nd^ Edition. (Prentice Hall College Div, 2000).

[11] Joint Institute for the Study of the Atmosphere and Ocean (JISAO), Pacific Decadal Oscillation (PDO)Index. http://research.jisao.washington.edu/pdo/. (2005), (Date of access: 30/08/2016).

[12] National Oceanic and Atmospheric Administration, How is the Polar Vortex Related to the Arctic Oscillation? https://www.climate.gov/news-features/event-tracker/how-polar-vortex-related-arctic-oscillation. (2014), (Date of access: 23/08/2016).

[13] Zhou S, Miller A, Wang J, Angell J (2001). Trends of NAO and AO and their associations with stratospheric processes. Geophys. Res. Lett..

[14] Jia B, Wang Y, Yao Y, Xie Y (2015). A new indicator on the impact of large-scale circulation on wintertime particulate matter pollution over China. Atmos. Chem. Phys..

[15] Ding YH, Liu YJ (2014). Analysis of long-term variations of fog and haze in China in recent 50 years and their relations with atmospheric humidity. Sci. China Earth Sci..

[16] Vautard R, Cattiaux J, Yiou P, Thépaut J, Ciais P (2010). Northern Hemisphere atmospheric stilling partly attributed to an increase in surface roughness. Nature Geosci.

[17] Seidel DJ, Fu Q, Randel WJ, Reichler TJ (2008). Widening of the tropical belt in a changing climate. Nature Geosci..

[18] McElroy M, Lu X, Nielsen C, Wang Y (2009). Potential for Wind-Generated Electricity in China. Science.

[19] Lu X, McElroy M, Sluzas N (2011). Costs for Integrating Wind into the Future ERCOT System with Related Costs for Savings in CO2 Emissions. Environmental Science & Technology.

[20] Lu, X., McElroy, M. and Sluzas, N. Costs for Integrating Wind into the Future ERCOT System with Related Costs for Savings in CO2 Emissions. *Environmental Science & Technology* **45**(7), pp.3160–3166. (2011). 10.1021/es103948t 21375280

[21] Lu X, McElroy M, Chen X, Kang C (2014). Opportunity for Offshore Wind to Reduce Future Demand for Coal-Fired Power Plants in China with Consequent Savings in Emissions of CO2. Environmental Science & Technology.

[22] Lu, X., McElroy, M., Chen, X. and Kang, C. Opportunity for Offshore Wind to Reduce Future Demand for Coal-Fired Power Plants in China with Consequent Savings in Emissions of CO2. *Environmental Science & Technology* **48**(24), pp.14764–14771 (2014). 10.1021/es503767x 25409413

[23] Fu G (2010). Temporal variation of wind speed in China for 1961–2007. Theoretical and Applied Climatology.

[24] Yang, X. Q., & Zhu, Y. M. Chapter 3 - Interdecadal climate variability in China associated with the Pacific decadal oscillation in *Regional Climate Studies of China*, 97–118 (Springer, 2008).

[25] Zhao S, Li J, Sun C (2016). Decadal variability in the occurrence of wintertime haze in central eastern China tied to the Pacific Decadal Oscillation. Sci. Rep..

[26] Wu B, Wang J (2002). Winter Arctic Oscillation- Siberian High and East Asian Winter Monsoon. Geophys. Res. Lett..

[27] Chen W, Feng J, Wu R (2013). Roles of ENSO and PDO in the Link of the East Asian winter monsoon to the following summer monsoon. J Clim.

[28] Xu M (2006). Steady decline of east Asian monsoon winds, 1969–2000: Evidence from direct ground measurements of wind speed. J. Geophys. Res..

[29] Keane A (2011). Capacity value of wind power. IEEE Transactions on Power Systems.

[30] Foley AM, Leahy PG, Marvuglia A, McKeogh EJ (2012). Current methods and advances in forecasting of wind power generation. Renewable Energy.

[31] Riddle EE, Butler AH, Furtado JC, Cohen JL, Kumar A (2013). CFSv2 ensemble prediction of the wintertime Arctic Oscillation. Climate dynamics.

[32] Kang D (2014). Prediction of the Arctic Oscillation in boreal winter by dynamical seasonal forecasting systems. Geophysical Research Letters.

[33] Buizer J, Jacobs K, Cash D (2016). Making short-term climate forecasts useful: Linking science and action. Proceedings of the National Academy of Sciences.

[34] Box, G. E. P., Jenkins, G. M. & Reinsel, G. C. Time Series Analysis: Forecasting and Control, 4th ed. (Wiley, 2013).

[35] Lapp SL, St. Jacques JM, Barrow EM, Sauchyn DJ (2012). GCM projections for the Pacific Decadal Oscillation under greenhouse forcing for the early 21st century. Int. J. Climatol..

[36] Visbeck M, Hurrell J, Polvani L, Cullen H (2001). The North Atlantic Oscillation: Past, present, and future. Proceedings of the National Academy of Sciences.

[37] Cane M (2005). The evolution of El Niño, past and future. Earth and Planetary Science Letters.

[38] Pryor SC, Barthelmie RJ (2011). Assessing climate change impacts on the near-term stability of the wind energy resource over the United States. Proceedings of the National Academy of Sciences.

[39] Rienecker MM (2008). The GEOS-5 data assimilation system-documentation, Versions 5.0.1, 5.1.0, and 5.2.0. NASA.

[40] Archer CL, Jacobson MZ (2005). Evaluation of global wind power. J Geophys Res: Atmos.

[41] Lu X, McElroy MB, Nielsen CP, Chen X, Huang J (2013). Optimal integration of offshore wind power for a steadier, environmentally friendlier, supply of electricity in China. Energy Policy.

[42] Gold Wind Company., GoldWind 1.5 MW Series Wind Turbine. http://www.goldwindamericas.com/sites/default/files/Goldwind-Brochure-1.5-Web.pdf. (2016), (Date of access: 30/09/2016).

[43] NASA Land Processes Distributed Active Archive Center (LP DAAC). MCD12C1: Land Cover Type Yearly L3 Global 500 m SIN Grid. https://lpdaac.usgs.gov/dataset_discovery/modis/modis_products_table/mcd12c1 (2012), (Date of access: 02/10/2016).

[44] Jarvis, A., Reuter, H. I., Nelson, A. & Guevara, E. Hole-filled seamless SRTM dataV4. http://srtm.csi.cgiar.org. (2008), (Date of access: 06/10/2016).

[45] Davidson M, Zhang D, Xiong W, Zhang X, Karplus V (2016). Modelling the potential for wind energy integration on China’s coal-heavy electricity grid. Nature Energy.

[46] GISS Surface Temperature Analysis (GISTEMP) Team, NASA Goddard Institute for Space Studies. http://data.giss.nasa.gov/gistemp/. (2016), (Date of access: 01/09/2016).

[47] Hansen, J., Ruedy, R., Sato, M. & Lo, K. Global surface temperature change. Rev. Geophys., **4**8, RG4004 (2010).

[48] Rohde R (2013). A New Estimate of the Average Earth Surface Land Temperature Spanning 1753 to 2011. Geoinfor Geostat: An Overview.

[49] National Oceanic and Atmospheric Administration. Pacific Decadal Oscillation (PDO). https://www.ncdc.noaa.gov/teleconnections/pdo/. (2016), (Date of access: 19/08/2016).

[50] National Oceanic and Atmospheric Administration. *Arctic Oscillation* (*AO*). https://www.ncdc.noaa.gov/teleconnections/ao/. (2016), (Date of access: 19/08/2016).

[51] National Oceanic and Atmospheric Administration. *Southern Oscillation Index* (*SO*). https://www.ncdc.noaa.gov/teleconnections/soi/. (2016), (Date of access: 19/08/2016).

