# Supplementary material for: Wind-generated Electricity in China: Decreasing Potential, Inter-annual Variability and Association with Changing Climate
Source: Sci Rep. 2017 Nov 24;7:16294. doi: 10.1038/s41598-017-16073-2 (PMC5701265; doi:10.1038/s41598-017-16073-2)
Supplement: Supplementary file 1 — Supplementary Information [file 41598_2017_16073_MOESM1_ESM.doc]

**Supplementary Information for** **Wind-generated Electricity in China: Decreasing Potential, Inter-annual Variability and Association with Changing Climate**

**Authors**

Peter Sherman^1^, Xinyu Chen^2*^ and Michael B. McElroy^1,2*^

**Affiliations**

^1^Department of Earth and Planetary Sciences, Harvard University, Cambridge, Massachusetts 02138, United States

^2^School of Engineering and Applied Sciences and Harvard China Project, Harvard University, Cambridge, Massachusetts 02138, United States

***Corresponding Author:** Michael B. McElroy (email: [mbm@seas.harvard.edu](mailto:mbm@seas.harvard.edu) , phone: 6174954359), Xinyu Chen (email: [xchen@seas.harvard.edu](mailto:xchen@seas.harvard.edu), phone: 6173190952

Province abbreviations

| **Abbreviation** | **Full name** |
| --- | --- |
| EIM | Eastern Inner Mongolia |
| GS | Gansu |
| HB | Hebei |
| HLJ | Heilongjiang |
| JL | Jilin |
| LN | Liaoning |
| SD | Shandong |
| WIM | Western Inner Mongolia |
| XJ | Xinjiang |


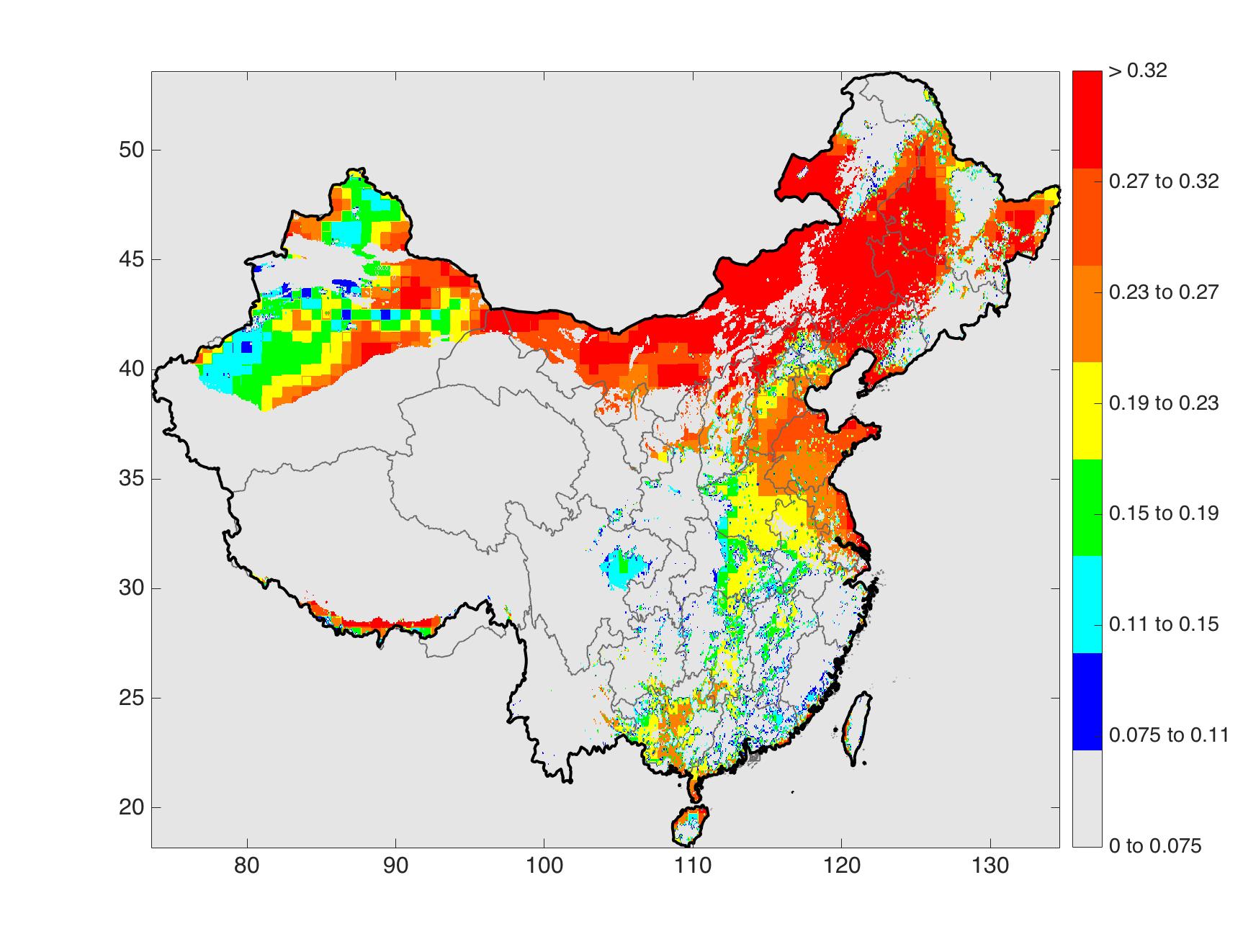
**Model comparison**

**Figure S1.** Spatial distribution of mean capacity factor for the geographically filtered regions over the period 2004 to 2008 based off the MERRA database. (This map is produced in Matlab R2014a.)

The MERRA wind data employed in this study were compared with wind data from MERRA-2 [S1] and NCEP/NCAR [S2] to assess consistency with the declining trend reported in this paper. Normalized annual average wind speeds over mainland China from the three datasets are plotted in Figure S2. Similar features can be seen in the individual time series, particularly with respect to the peaks in wind speed observed around 1984, 1996 and 2009, an indication of consistency between the three datasets. Most important, for purposes of this paper, is the declining trend indicated with each dataset. The decline from the MERRA data (-1.08 ± 0.38E-3) agrees with the indication from NCEP/NCAR (-1.27 ± 0.53E-3). Consistency between the two datasets provides support for the declining trend reported in the paper.


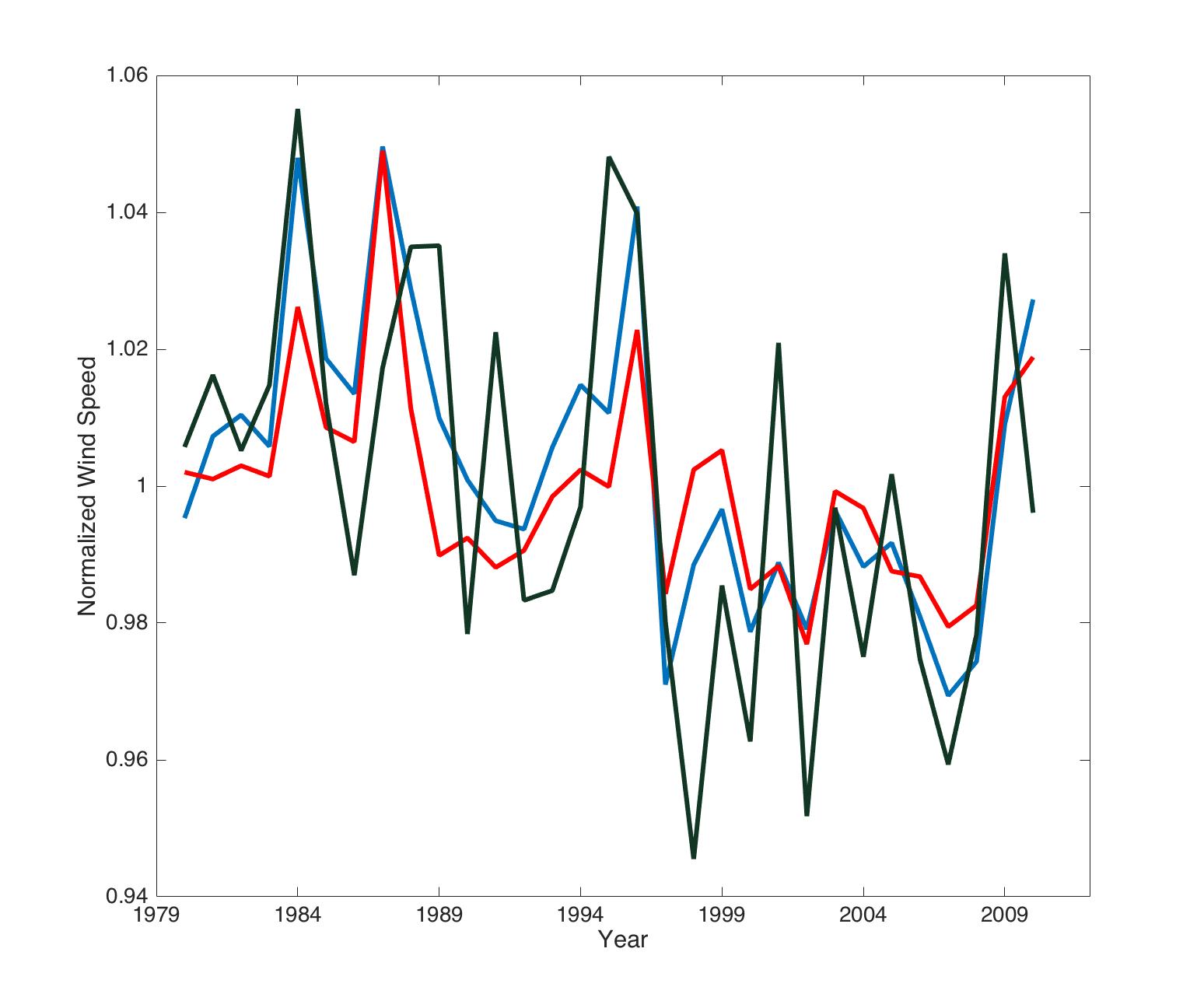


**Figure S2.** Time series of normalized wind speed over China for the MERRA (red), MERRA-2 (blue) and NCEP/NCAR (green) datasets. The normalized wind speed is defined as the annual average wind speed divided by the average value over the time span.

**Monthly variation**

To observe the monthly variation in wind production, the mean monthly variation in capacity factor was calculated and shown in Figure S3. This calculation was done over only Western Inner Mongolia because this area shows the greatest variation between seasons and contains the largest number of wind bases. A peak can be seen in April followed by a trough in the summer months. The average capacity factor for wind power reached 0.46 in March and April, 44% higher than the average for July and August. It clearly illustrates that electricity generation from wind is strongest in winter and spring months; the influence of summer months is much less significant.


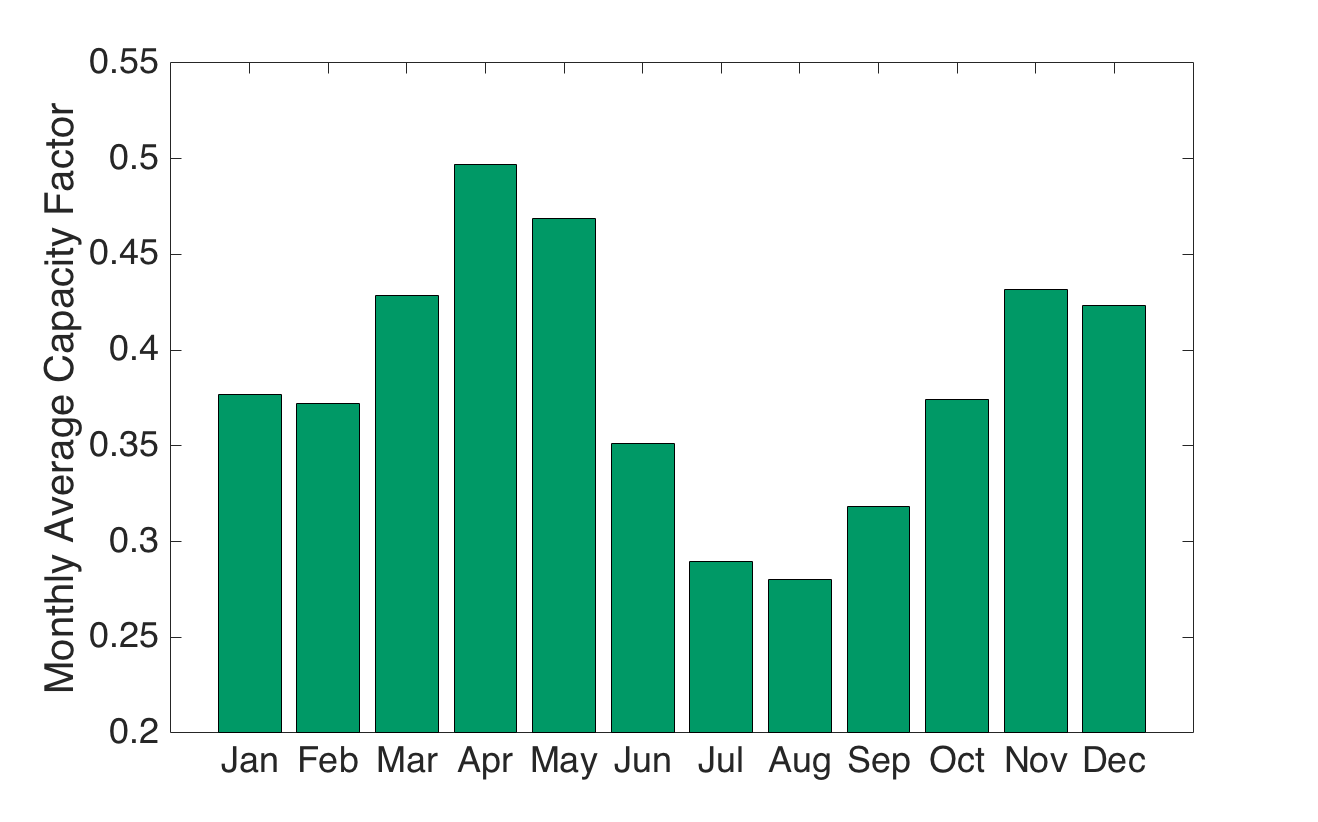


**Figure S3.** The mean monthly variation in capacity factor over Western Inner Mongolia during the studied 37 year period.

**Multi-variable regression model**

The regression model defined by equation 1 was applied to annual mean values of CF derived for the region as a whole and for three particularly important provinces: Hebei, western Inner Mongolia, and Xinjiang. Regression coefficients for each of the variables, a_1_-a_5_, are summarized in Table S1. With four of the coefficients associated with the climate variables PDO, AO, SO and time, with a total of 37 values of CF (one for each year included in the fitting procedure), the number of degrees of freedom is equal to 32.

**Table S1. Regression modelling coefficients.** Coefficients employed in the regression models (see defining equation in text)

| **Coefficients w/ all** | **Hebei** | **Western Inner Mongolia** | **Xinjiang** | **Entire Analyzed Region** |
| --- | --- | --- | --- | --- |
| **a_1_** | 1.32 | 3.88 | 1.55 | 2.08 |
| **a_2_** | -1.54E-5 | -3.33E-3 | 4.71E-3 | -1.83E-3 |
| **a_3_** | -2.09E-2 | -3.76E-2 | -9.29E-3 | -1.45E-2 |
| **a_4_** | -3.30E-3 | 4.07E-4 | -6.39E-4 | -1.29E-3 |
| **a_5_** | -5.21E-4 | -1.75E-3 | -1.68E-3 | -8.79E-4 |

The regression models with different combinations of input variables were compared on the basis of AIC criteria. The AIC values for regression models with any combination of variables are summarized in Table S2. For regions of Hebei, West Mongolia and for the entire region, the optimal model would incorporate only the AO index, since the corresponding AIC value is lowest. The AIC value for the model employing PDO, AO and SO indices is, however, close to this optimal value. For Xinjiang, the best regression model in terms of AIC values would employ both the PDO and AO indices. The optimal parameters for the regression model are summarized in Table S3.

Note that the optimal combination of input parameters for the regression model may be different in the forecasting procedure especially when the AIC values for different models are relatively close, since the state-of-art forecasting accuracies for the PDO and SO are significantly better than for the AO.

**Table S2. AIC values.** AIC values of the regions analyzed for each set of oscillation parameters. The set of parameters used in each regression model is highlighted.

| **Region** | **PDO, t** | **PDO, AO, t** | **PDO, SO, t** | **PDO, AO, SO, t** | **AO, SO, t** | **AO, t** | **SO, t** |
| --- | --- | --- | --- | --- | --- | --- | --- |
| **Hebei** | -209.5 | -224.9 | -207.6 | -223.7 | -225.7 | -226.6 | -208.6 |
| **West Inner Mongolia** | -166.2 | -184.3 | -165.5 | -182.3 | -183.8 | -185.4 | -167.2 |
| **Xinjiang** | -209.6 | -210.4 | -207.6 | -208.5 | -208.7 | -208.6 | -206.4 |
| **Whole Region** | -224.2 | -234.4 | -222.5 | -232.6 | -234.0 | -236.0 | -224.4 |

**Table S3. Regression modelling coefficients using AIC criteria.** Coefficients employed in the regression models displayed in Figure 6 (after accounting for the AIC criteria)

| **AIC Coefficients** | **Hebei** | **Western Inner Mongolia** | **Xinjiang** | **Entire Analyzed Region** |
| --- | --- | --- | --- | --- |
| **a_1_** | 1.42 | 3.60 | 1.55 | 1.97 |
| **a_2_** | 0 | 0 | 5.47E-3 | 0 |
| **a_3_** | -2.03E-2 | -3.69E-2 | -8.78E-3 | -1.38E-2 |
| **a_4_** | 0 | 0 | 0 | 0 |
| **a_5_** | -5.73E-4 | -1.61E-3 | -6.36E-4 | -8.25E-4 |

Note: the regression coefficient is set equal to 0 if the variable is rejected on the basis of the AIC test.

**Table S4. Regression model fits.** r^2^ and adjusted r^2^ values for the regression model coefficients presented in Tables S1 and S3.

| **Region** | **AIC Adjusted r^2^** | **AIC r^2^** | **Adjusted r^2^ w/ all** | **r^2^ w/ all** |
| --- | --- | --- | --- | --- |
| **Hebei** | 0.48 | 0.51 | 0.46 | 0.52 |
| **West Inner Mongolia** | 0.60 | 0.62 | 0.59 | 0.63 |
| **Xinjiang** | 0.38 | 0.43 | 0.36 | 0.43 |
| **Whole Region** | 0.55 | 0.57 | 0.53 | 0.58 |

**Multi-variable regression model for de-trended wind capacity factors**

To examine the effectiveness of the proposed regression model to account for the natural oscillations in representing the variation of wind power, we removed the declining trend in the annual average capacity factor and conducted the same regression analysis. The regression model employs possible combinations of the PDO, AO and SO as input variables and evaluates their effectiveness with the AIC criteria as implemented previously. The best fit employs the PDO and AO as variables, with corresponding results displayed in Figure S4. The r^2^ value of this model is 0.42. The model still produces a good fit for the inter-annual variation in de-trended wind CF.


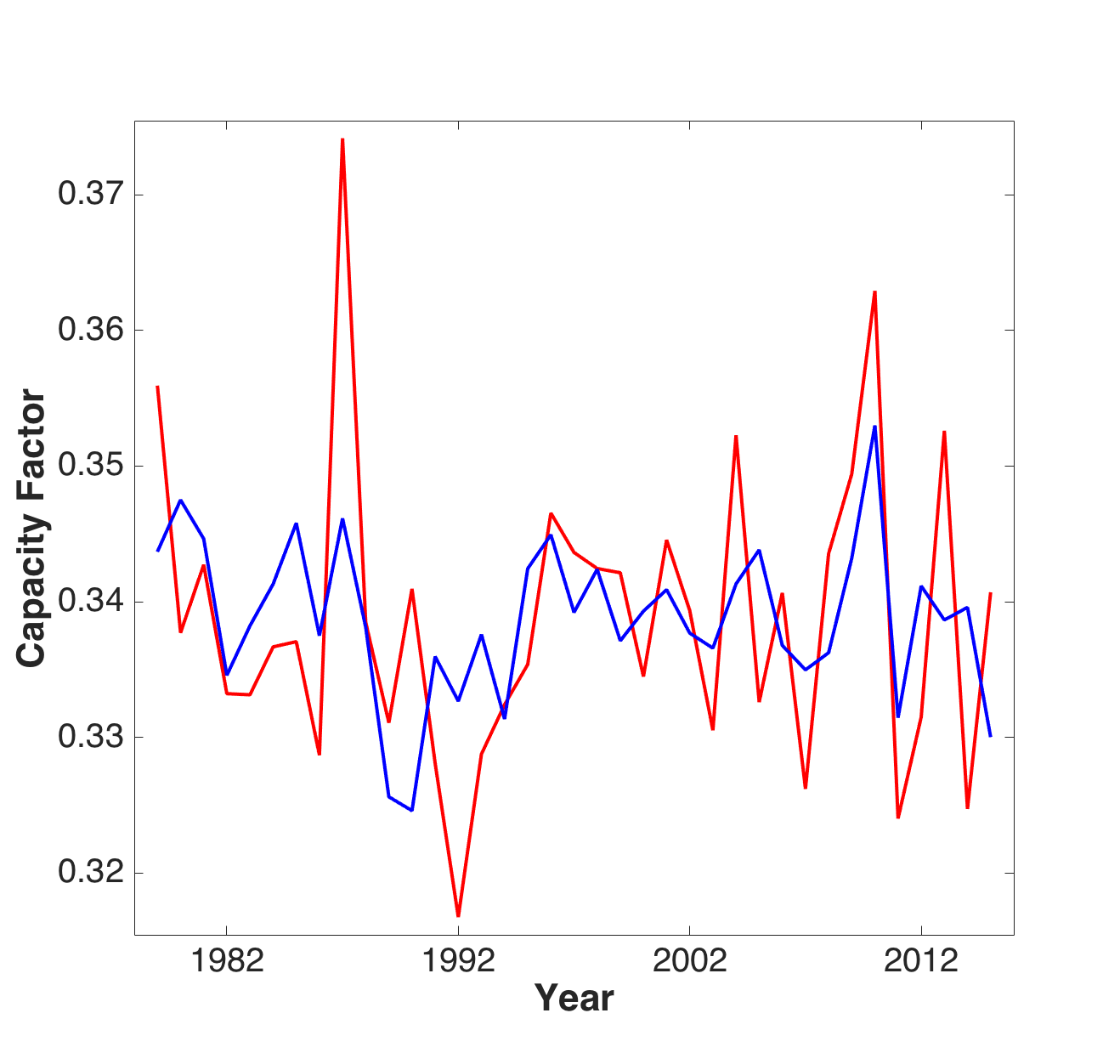


**Figure S4.** A time series for de-trended values of CF derived for the entirety of the analyzed region is shown in red. Results obtained using the regression model are indicated in blue.

**References**

**[S1]** The Modern-Era Retrospective Analysis for Research and Applications, Version 2 (MERRA-2), Ronald Gelaro, et al., 2017, J. Clim., doi: 10.1175/JCLI-D-16-0758.1

**[S2]** Kalnay, E., M. Kanamitsu, R. Kistler, W. Collins, D. Deaven, L. Gandin, M. Iredell, S. Saha, G. White, J. Woollen, Y. Zhu, M. Chelliah, W. Ebisuzaki, W. Higgins, J. Janowiak, K.C. Mo, C. Ropelewski, J. Wang, A. Leetmaa, R. Reynolds, R. Jenne, and D. Joseph, 1996: The NCEP / NCAR 40-year reanalysis project. *Bull. Amer. Meteor. Soc.*, **77**, 437-471.
